# Label-Free Enrichment of Circulating Tumor Plasma Cells: Future Potential Applications of Dielectrophoresis in Multiple Myeloma

**DOI:** 10.3390/ijms231912052

**Published:** 2022-10-10

**Authors:** Nicolò Musso, Alessandra Romano, Paolo Giuseppe Bonacci, Grazia Scandura, Clarissa Pandino, Massimo Camarda, Giorgio Ivan Russo, Francesco Di Raimondo, Emma Cacciola, Rossella Cacciola

**Affiliations:** 1 Department of Biomedical and Biotechnological Sciences (BIOMETEC), University of Catania, 95125 Catania, Italy; 2 StLab SRL, 95126 Catania, Italy; 3 Department of General Surgery and Surgical Medical Specialties, University of Catania, 95125 Catania, Italy; 4 Urology Section, University of Catania, Via S. Sofia 78, 95125 Catania, Italy; 5 Department of Medical, Surgical Sciences and Advanced Technologies “G.F. Ingrassia”, University of Catania, 95123 Catania, Italy; 6 Hemostasis/Hematology Unit, A.O.U. Policlinico “G. Rodolico-San Marco”, 95123 Catania, Italy; 7 Department of Clinical and Experimental Medicine, University of Catania, 95123 Catania, Italy

**Keywords:** minimal residual disease, multiple myeloma, acute leukemia, lymphoma

## Abstract

In multiple myeloma (MM), circulating tumor plasma cells (CTPCs) are an emerging prognostic factor, offering a promising and minimally invasive means for longitudinal patient monitoring. Recent advances highlight the complex biology of plasma cell trafficking, highlighting the phenotypic and genetic signatures of intra- and extra-medullary MM onset, making CTPC enumeration and characterization a new frontier of precision medicine for MM patients, requiring novel technological platforms for their standardized and harmonized detection. Dielectrophoresis (DEP) is an emerging label-free cell manipulation technique to separate cancer cells from healthy cells in peripheral blood samples, based on phenotype and membrane capacitance that could be successfully tested to enumerate and isolate CTPCs. Herein, we summarize preclinical data on DEP development for CTPC detection, as well as their clinical and research potential.

## 1. Introduction

Multiple myeloma (MM) is a blood clonal B-cell malignancy due to an uncontrolled proliferation of plasma cancer cells (PCs) in the bone marrow (BM) or, more rarely, in extramedullary tissues, which is characterized by the presence of a monoclonal component (M protein) in serum and/or urine, composed of immunoglobulins produced by cancer cells [1]. Neoplastic PCs proliferate within a niche microenvironment, which may be in the bone marrow itself, or outside, providing the biological basis of spatial and temporal heterogeneity in MM [2].

Thanks to recent advances in immune chemotherapy (due to the introduction of CAR-T cells, bispecific antibodies and monoclonal antibodies), the overall survival increased in the last decade [3], but most patients still relapse, resulting in shorter and shorter remissions [3], due to the emergence of new subclones with variable sensitivity to drugs and dependence on the microenvironment support signaling [4]. However, staging and follow-up monitoring are still largely based on evaluation of tumor plasma cells (TPCs) in the bone marrow (BM), quantified via an integrative approach based on flow cytometry, morphology assessment and molecular biology [3,5,6].

After treatment, the most relevant prognostic factor is the achievement of negative minimal residual disease (MRD), defined by the absence of plasma cells in the bone marrow by NGS or NGF, by next-generation flow cytometry (NGF) as well as with next-generation sequencing technologies (NGS), two methods that have a minimum sensitivity of 1 in $10^{6}$ nucleated cells in patients with complete remission and confirmed at least 1 year apart to be “sustained”, regardless of treatment, cytogenetic risk and ISS stage [6,7]. Unfortunately, both NGS and NGF are expensive, time consuming and require many BM cells (at least 1.5 × 10^6^), challenging the longitudinal sampling during follow-up, especially for elderly patients and for those treated intensively who can develop bone marrow hypoplasia. To overcome these limitations and the spatial clonal heterogeneity, typical of MM, new approaches have been explored, including coupling imaging techniques, such as PET or MRI to free circulating DNA (cfDNA) using NGS [8], well described in other relevant reviews, or to quantify circulant tumor plasma cells (CTPCs). Reflecting the whole tumor heterogeneity, CTPCs could be used as diagnostic markers [8] and represent a promise for MM response monitoring, being able to be analyzed by molecular approaches [9,10].

In this manuscript, we will review the technological platforms available to detect CTPCs in peripheral blood of MM patients, with a major focus on novel technologies based on dielectrophoresis coupled to microfluidics to warrant downstream genomic and functional assays.

## 2. The Emerging Prognostic Role of CTCs in Multiple Myeloma

### 2.1. Clinical Relevance of CTC Detection in Multiple Myeloma

Physiologically, PCs exhibit a unique migration pattern, characterized by intermittent periods of high motility and longer stretches of confined migration or arrest. Long-lived PCs migrate and cluster in the BM, to better access to survival soluble factors released by stromal cells, such as APRIL, as recently shown in vivo by time-lapse intra-vital BM imaging [11,12]. In MM, CTPCs can be found in peripheral blood as a consequence of the complex spatiotemporal interaction [13] with the pro-inflammatory and hypoxic BM microenvironment, which causes arrest in proliferation, enhancing trafficking and forcing TPCs to recirculate. Indeed, differently from neoplastic PCs in the bone marrow, CTPCs in MM are mostly apoptopic (arrested in the G0–G1 phase of the cell cycle) [14], with a significantly lower proliferation index than their BM counterpart, following a circadian rhythm similar to CD34+ cells [15]. Thus, CTPCs are highly informative about both intra and extramedullary disease at the phenotypic, genomic and transcriptomic levels [16].

CTPCs have successfully been quantified in peripheral blood (PB) of MM patients. Pilot studies showed that MM patients carrying CTPCs within the range of MGUS patients had significantly longer PFS and OS, independent of the response to therapy evaluated according to both the response and MRD status. The persistence/presence of CTPCs in MM patients who had undergone therapy might be used as a surrogate marker of BM MRD positivity, since all treated MM patients who showed CTPCs after therapy always showed MRD positivity in paired BM samples [17].

Two recent prospective large studies confirmed the prognostic relevance of CTPCs in MM. First, in 374 newly diagnosed MM patients enrolled in two clinical trials (GEM2012MENOS65 and GEM2014MAIN) who received first-line treatment based on bortezomib, lenalidomide and dexamethasone induction followed by autologous stem cell transplantation, consolidation and maintenance, CTPCs were detected by NGF in more than 90% of newly diagnosed MM patients [18]. Despite the correlation between the percentages of CTPC and BM PCs being modest, increases in logarithmic percentages of CTPCs (with a cutoff of 0.01%) were associated with inferior progression-free survival (PFS), independently from the International Staging System, lactate dehydrogenase levels and cytogenetics. Outcomes according to percentage of CTPCs and depth of response to treatment showed that 90% of patients with undetectable CTPCs remained progression free after a median follow-up of 5 years, regardless of complete remission and measurable residual disease (MRD) status. Among all patients with detectable CTPCs, only achieving MRD negativity—not complete remission—was associated with significant improvement in progression-free survival [18]. Second, in 401 newly diagnosed MM patients randomized in the FORTE clinical trial to receive carfilzomib, cyclophosphamide and dexamethasone or lenalidomide and dexamethasone followed by autologous stem cell transplantation, a cutoff of 0.07% CTPC identified patients with shorter PFS and overall survival (OS) [19].

### 2.2. Platforms Available to Detect CTPCs in Multiple Myeloma

Nowadays, there is no gold standard for detecting and counting circulating tumor cells (CTCs), but there are two competing modes available, label based and label free. Each technique has advantages and disadvantages, while their combination can allow for a more complete and exhaustive characterization [20,21], as summarized in Table 1.

Label-based (or affinity-based) capture is the most widely used strategy, distinguished into magnetically activated cell sorting (MACS) and fluorescence-activated cell sorting (FACS), after the application of multiparametric flow cytometry (MFC) or next-generation flow (NGF) analysis. Label-free detection methods can be based on cell size, deformability, density and electrical differences.

#### 2.2.1. Immune Phenotype of CTPCs

MFC is a staining technique that uses different markers’ clonal plasma cells with an aberrant immune phenotype, developed for the detection of minimal residual disease (MRD) in the BM [7,22,23]. The most common aberrancies examined are loss of CD19 expression, decreased expression of CD45, gain of CD56 and aberrant CD117 expression. The immune phenotype of CTPCs differs from the BM-PCs counterpart for downregulation of integrins (CD11a/CD11c/CD29/CD49d/CD49e), adhesins (CD33/CD56/CD117/CD138) and activation molecules (CD28/CD38/CD81), which specifically characterizes circulating neoplastic PCs, reflecting how hypoxic and pro-inflammatory microenvironment could induce an arrest in proliferation, forcing tumor cells to recirculate in peripheral blood [23].

However, lack of standardization and multi-color instrumentation to assess the expression of more markers simultaneously limited, in the past, the use of MFC to detect CTPCs in daily clinical practice. In the last few years, applying an NGF approach, based on processing at least 10 × 10^6^ PB cells/tube stained with the two-tube/eight-color EuroFlow-IMF MM MRD antibody panel, the limit of detection can be set at 20 tumor plasma cell events, resulting in ~two-fold increased frequency of cases presenting with CTPC in PB by NGF vs. both immunocytochemistry and conventional flow cytometry, among MGUS, smoldering and active MM [23,24].

Use of new high throughput can improve sensitivity but it is needed to manage the phenotype heterogeneity and low numbers requiring harmonization for fluorochrome panel design, sample processing and data analysis, possibly by integrating means of artificial intelligence and dedicated pipelines. In particular, the introduction of anti-CD38 immunotherapy in the upfront treatment of most patients challenges the CTPC identification via CD38 expression, due to either masking of the CD38 epitope(s) targeted by flow cytometric antibodies or, less commonly, downregulation of CD38 expression by the neoplastic plasma cell clone through natural selection. For this reason, additional markers (such as CD54 (ICAM-1), CD229 (SLAMF3), CD269 (BCMA), CD319 (SLAMF7) and VS38c can be required, challenging the gating analysis and requiring computational approaches, such as Flow-CT, developed for MRD detection or large immune monitoring studies, to perform quality control and analyze high-dimensional data [25].

#### 2.2.2. Liquid Biopsy to Analyze ctDNA and cfDNA by NGS

Two pilot studies investigated the role of liquid biopsy for CTC in PB by analyzing ctDNA using NGS [26,27]. The aim of these studies was to assess the rearrangement of the IgH gene within ctDNA, using an NGS approach based on amplicons BIOMED2-FR1/-FR3 (IgH), primer pool -Ig kappa (IGk) or -Ig lambda (IGl), both at diagnosis and after treatment.

Genomic features of matched PCs isolated from BM and PB samples showed that ~20% of CTPCs egressed from a site distant from the matched BM aspirate, with high concordance between BM and PB plasma cells for chromosome arm-level copy number alterations (≥95%) but not for translocations (39%). Using ultra-low pass whole-genome sequencing (ULP-WGS), CTPCs can capture the genetic diversity of matched tumor biopsies, leading to identification of subclones not detectable in the bone marrow. However, the major limitation to the genomic evaluations for CTPCs was the enrichment step, which could result in substantial carryover of white blood cells and affect the ability to detect limited numbers of CTPCs [16].

All high-risk genetic abnormalities, except one t(4; 14), were detected in CTPCs whenever present in BM tumor cells. More than 80% of the mutations present in plasma cells sorted from bone marrow and extramedullary sites were detectable in CTPCs [28].

#### 2.2.3. Limitations for Clinical Application of CTPC Detection in Multiple Myeloma

Major limitation in CTC detection is the low number, usually equal to 5–50 CTCs for every 7.5 mL of peripheral blood in a patient with metastatic cancer, leading to a 10^−5^ sensitivity. Since neoplastic cells are quantified in relation to other cells in the sample, the smallest cluster of abnormal cells that can be reliably called CTCs depends on the total number of cells analyzed. With 2–5 × $10^{5}$ cells acquired, a cluster of 20–50 cells represents a sensitivity of 1 in $10^{4}$ (0.01%). The smaller is the number of abnormal cells present so the larger must be the number of cells that needs to be analyzed. Another issue limiting the CTC detection is their downstream manipulation after enrichment, requiring a dedicated workflow to warrant cell integrity and viability [29].

In solid tumors, diagnostic leukapheresis (DLA) has currently been validated by the European consortium CT Trap and UDUS but requires the processing of 2.5 L of blood to increase the yield of CTCs by up to 100-fold compared to the volume of a normal blood sample of about 10 mL [30]. Through DLA, CTCs can be identified more frequently and reliably in patients with metastases from solid tumors, without significant side effects, an approach never tested for liquid tumors. However, the bottleneck of DLA is the high background of co-isolated white blood cells, which limits the use of complete DLA products.

To overcome the main limitations in CTC detection, novel technological platforms are required to improve their enrichment by increasing sensitivity, specificity and cell integrity for downstream processing [31], through different detection platforms, such as fluorescence and electrochemical detection. To this end, microfluidics-based technologies could allow the separation of CTCs from whole blood, isolating cells and analyzing them from different perspectives, such as morphology, genomics, proteomics, transcriptomics and other cell biological functions [32].

An example of a very promising microfluidic technology is the CROSS chip [33], a cellular filter that allows for the label-free isolation of CTCs from 7.5 mL of whole blood, obtained from colorectal cancer patients, based on different cell sizes and deformability.

The inertial, label-free cell dielectrophoresis sorting, described in the next section, stems from differences in the dielectric properties, as well as size and physical geometry of CTCs and other blood cells; thus, separation can be achieved by applying the appropriate electrical wave frequency [34].

## 3. Dielectrophoresis (DEP)

### 3.1. DEP: Phisical Principles

Dielectrophoresis is a label-free technique defined by Herbert Pohl in 1950 as the translational movement of a polarizable particle in a non-uniform electrical field [35].

**Figure 1:**
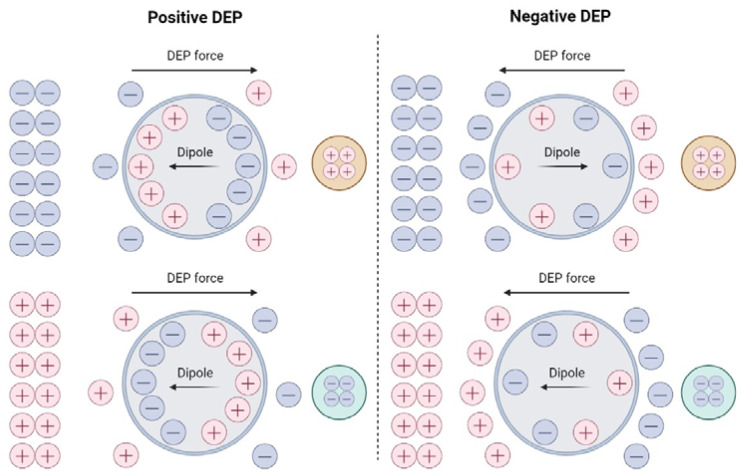
Polarization induced net dipole and dielectrophoretic force direction for a particle that is (**left**) more polarizable than the surrounding medium and (**right**) less polarizable. Top and bottom are two opposite arrangements of the background electric field.

The lipid membrane of the cell serves as an insulating (dielectric) layer between the conductive cytoplasm and the aqueous environment. When cells are placed in an ionic medium and exposed to an electric field, the charges within the particle can be rearranged near the plasmatic membrane, which is the interface between the particle and the medium, producing an electric dipole (Figure 1). If a nonuniform electric field is applied, a force is generated to move or suspend the cell, the basic physical principle used in cell DEP.

**Figure 2:**
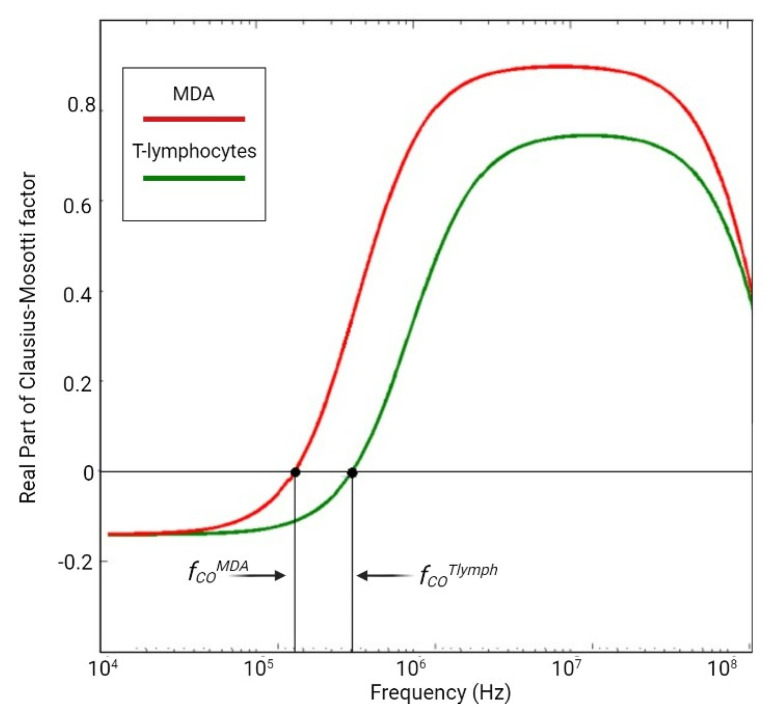
Clauss-Mosotti function for MDA, a CTC derived from breast cancer, versus healthy cells in peripheral blood.

When the external electric field is non-uniform, cells are subjected to opposite forces so their movement depends on the resulting net force, called the dielectrophoretic (DEP) force, which is measured by an equation that refers to the simplifying case of a spherical particle [36,37]:

(1) $$\langle F_{DEP}\rangle =2\pi R\varepsilon_{m}f_{CM}\nabla {\left|E_{rms}\right|}^{2}$$

where *R* is the radius of the spherical particle, *ε_m_* is the relative permittivity of the surrounding medium, *f_CM_* represent the real part of Clausius–Mossotti (Figure 2) factor and ∇|*E*| is the amplitude of the electric field.

In particular, *f_CM_* factor is defined by the following equation:

(2) fCM=Re[εp*−εm*εp*+2εm*]

including the complex permittivity of the particle (*ε_p_*) and the surrounding medium (*ε_m_*) [38,39,40,41], which represents the polarizability parameter, changing the function of the applied field frequency and in relation to the *ε_p_* and *ε_m_*. Thus, F_DEP_ direction depends on Re(*f_CM_*); if *ε_p_* > *ε_m_*, Re(*f_CM_*) is positive (Re(*f_CM_*) > 0) as also F_DEP_, vice versa when *ε_p_* < *ε_m_*, Re(*f_CM_*) is negative (Re(*f_CM_*) < 0) and also F_DEP_. Thus, particles with higher polarizability than the surrounding fluid are attracted toward the higher electric field strengths, positive DEP (pDEP) [41]. The transition from nDEP to pDEP (or pDEP to nDEP) occurs at a specific frequency defined as crossover frequency (*f_CO_*) [39,41]. At *f_CO_*, the complex permittivity of the particle and the surrounding medium are exactly equal, so the net DEP force acting on the particle is equal to zero [42].

When DEP is applied to mammalian cells, at *f_CO_*_1_, a transition from negative to positive DEP occurs, whilst at the much higher frequency *f_CO_*_2_, there is a transition back to negative DEP, since conductivity in cell cytoplasm is higher than the conductivity in the medium, as formalized in the following equation:

(3) $$f_{CO1}=\frac{\sigma_{m}}{R\varphi C_{0}}$$

where *σ_m_* is the medium conductivity, *R* the outer cell radius and *φC*_0_ refers to the capacitance per unit area of the smooth plasma membrane, determined as 0.009 F/m [43,44] and *φ* is the folding factor, characterizing the different membrane features, such as ruffles, folds and microvilli. *Rφ* can be considered as the “dielectric phenotype” of a given cell type, determining its response to DEP manipulation.

Thus, the low crossover frequency *f_CO_*_1_ is closely related to membrane capacitance and conductance, which reflects, respectively, its morphological complexity (as area, thickness, composition) and the presence of ion transporters across the membrane [45]. Ions, such as potassium, with different concentration gradients or the osmotic flux of water across the plasma membrane do not influence measurements of the lower-frequency DEP crossover [46], unless they lead to significant changes in cell volume, morphology or viability [43,44].

When the frequency becomes higher, the electric field starts penetrating inside the cell, thus, “probing” its content. High crossover frequency *f_CO_*_2_ depends on the effective internal cellular conductivity and permittivity, reflecting the electrical mobility of ion species and the combined properties of cytoplasmic water and intracellular barriers to charge movement. Thus, *f_CO_*_2_ is highly informative to determine cell viability, the cell-cycle phase and apoptosis, which lead to changes in the nucleus volume fraction [38,47], the chemical composition of cell suspending medium [48] to discriminate cells based on cytoplasm features [45,49]. Since the complexity of the cytoplasmic membrane is given by the presence of several proteins to maintain cell morphology and play physiological functions, their changes lead to a different response to fCO2 between healthy and malignant cells [47,48,49,50].

### 3.2. DEP to Separate Plasma Cells in Peripheral Blood

Every single cell has dielectric properties, letting DEP discriminate blood cells with a Δ diameter of only 4 μm, with a sensitivity of about 1000 cancer cells for every milliliter of blood [51,52].

**Figure 3:**
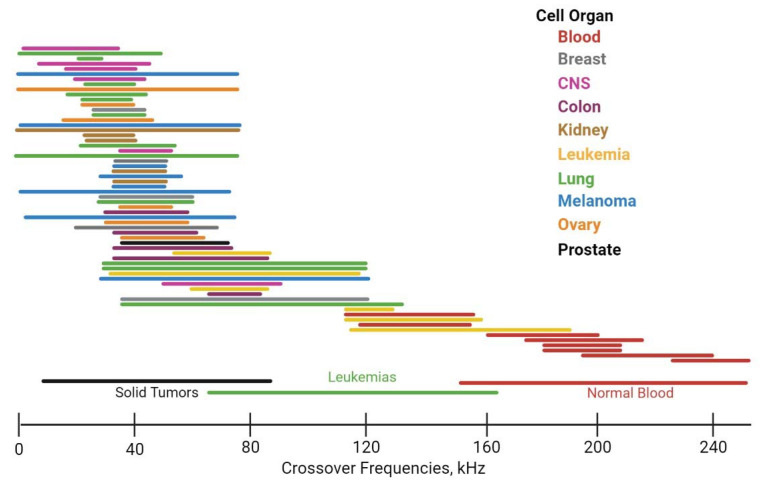
DEP responses of cancer and normal blood cells expressed in terms of first crossover frequency.

As shown in the widely representative NCI-60 panel of cancers [52], each cell type has different fCO so it is an essential parameter for cell separation (Figure 3), as a consequence of cell stiffness [53], which is highly correlated with biological functions. The mechanical properties of cells are closely related to several biological functions, such as cell differentiation, ageing, motility, metastasis, etc. For instance, metastatic cancer cells are softer than normal cells [54].

Dielectric properties of plasma cells can be theoretically derived by the multi-shell model, reflecting cell complexity given by plasma membrane, cytoplasm and nuclear membrane that are three homogenous dielectric regions with specific electrical properties [41,44,45].

Membrane electrical parameters of murine plasma cells have been known since 1991, with a membrane conductance of 380 S/m^−2^. The mean *f_CO_*_2_ value of 195 MHz reflects the unique morphology of a plasma cell, due to the anisotropy of the cell interior given by the nucleus, which occupies about 80% of the cytoplasmic volume cell and by the existence of cytoplasmic membrane-like material, such as organelles and endoplasmic reticulum. Using SP2/O murine myeloma cells showed that in DEP with low-voltage signals (from ~1 kHz to no higher than ~30 MHz), the value of crossover frequency is determined by the conductivity of the suspending medium, as well as the size and shape of the cell and the dielectric properties (capacitance, conductivity) of its plasma membrane [55]. While high-frequency DEP (voltage up to 400 MHz) showed a higher crossover frequency, which is predominantly determined by intracellular conductivity, particularly, it is correlated to that of the intracellular potassium concentration [56].

Under the same conditions, cancer cells exhibit consistently lower *f_CO_*_1_ values than normal peripheral blood cells [57,58], leading to the development of DEP as a label-free technique for isolating circulating tumor cells from peripheral blood.

However, cell DEP is a microscale phenomenon, which poses significant technical challenges when applied to large numbers of cells, such as peripheral blood requiring to be combined with microfluidics [36,58].

DEP can be combined with flow-through lateral field flow fractionation (DPE-LFFF) to separate CTCs [59] or platelets from blood cells [36], providing a very efficient separation coupled with high platelet/CTC purity (~99%) with almost no cell loss (<2%).

Another approach developed a DEP microfluidic device (H-DC-iDEP) based on hydrodynamic and direct current polydimethylsiloxane (PDMS) insulator that separates plasma from fresh blood [60] to study plasma, without the need for pre- or post-processing steps. Other applications of DEP microfluidic devices are the separation of different cell types, such as leucocytes (floating cells), based on different membrane size and properties and X and Y spermatozoa based on membrane charge and cytoplasm conductivity [61].

The value of fCO2 for cells suspended in DEP medium gradually decreased over time, for murine plasma cells at 19 MHz/h at 21 °C for the first two hours, limiting the time that the cells could be suspended and, consequently, the throughput. Moreover, the magnitude of the positive DEP force decreased steadily with time, leading to difficulties in clear observation of the attraction of cells to electrodes when DEP is combined with microfluidics [56,60].

### 3.3. DEP Applications in Cancer

Undifferentiated glioblastoma cells show much lower crossover frequencies than the fully differentiated cells cultured in normal conditions, due to a unique profile of intracellular dielectric characteristics reflecting the intrinsic biological properties, suggesting the potential role of DEP to monitor cell population at different stages of differentiation and the need of a repository of crossover frequencies to monitor individual patients.

Since multi-drug resistance is correlated with ion channel modulators in the cell membrane, with modulation of membrane-associated proteins as P-glycoprotein (P-gp) and multidrug-resistance-associated protein (MRP), different cell electrical properties are observed; DEP has been used to successfully separate drug-resistant cells from sensitive counterparts [62]. Indeed, the cytoplasmic conductivity of doxorubicin-resistant K562 (K562/doxR) cells was almost two-times higher than that of drug-sensitive K562 cells, as analyzed by DEP collection spectra, due to a lower membrane potential and a higher cytoplasmic conductivity [62]. Treatment with 30 nM of Doxorubicin, an anthracycline antibiotic commonly used in the treatment of hematopoietic and solid tumor, induced a reduction in *f_CO_*_1_ [63]. When cells died and underwent significant morphological changes, at DOX concentrations > 25 μM, the cell *f_CO_*_1_ increased dramatically, as a consequence of cell membrane alterations and mitochondrial membrane potential collapse, which may be the underlying mechanism behind the cell *f_CO_* shift [64].

Using a 3D-electrode contactless DEP device, it was possible to measure the *f_CO_*_2_ to identify K562 resistant to imatinib or doxorubicin and to trap them with higher efficiency when increasing the concentration of doxorubicin (up to 300 nM), obtaining a nonlinear equation that can be used to estimate the level of drug resistance in relation to trapped cells, with 100% selectivity in a mixture containing less than 1% of resistant cells [64,65,66]. In this way, DEP can be utilized for the detection of the level of resistance in K562 (doxo-resistant K562) cells without requiring the examination of P-gp dynamic functions, avoiding the use of invasive methods to isolate cancer cells [36].

However, current DEP systems suffer from critical limitations, mainly associated with their low throughput, which has hindered their standardization and widespread use [29]. When the cells cross the DEP device, it is necessary that they remain intact as regards the biological, biochemical and biophysical properties, in order to be subsequently manipulated and studied; therefore, cellular integrity must be preserved [29]. The causes that could lead to cell lysis are many, including excessive charge in the cell membrane in the electric field, suspension in a non-physiological buffer and shear stress induced by too fast a flow [67].

Another major concern is maintaining cell viability in lab-on-a-chip devices based on the DEP method overcoming Joule heating. Typically, the local electric field around the microelectrodes is relatively high and causes an electric current to pass through the conductive medium, resulting in significant heating of the medium and cells and denaturation of their proteins. Temperature gradients greater than 20 K are associated with irreversible cell damage [68]. Joule heating can be controlled and limited by optimizing the applied voltage, maintaining the medium’s relatively low electrical conductivity (generally below 100 mS/m) and configuring the electrodes in the coupled microfluidics [29].

The dielectroforetically activated cell sorting (DACS) method is an emerging cell separation technique associated with DEP [60]. Depending on the frequency of externally applied electric fields, DACS will depend mainly on membrane morphology (medium frequencies, 10–200 kHz) or cytoplasmic composition (high frequencies, 5–400 MHz) rather than on a cell’s phenotype as with FACS (fluorescence-activated cell sorting) and MACS (magnetic-activated cell sorting).

**Figure 4:**
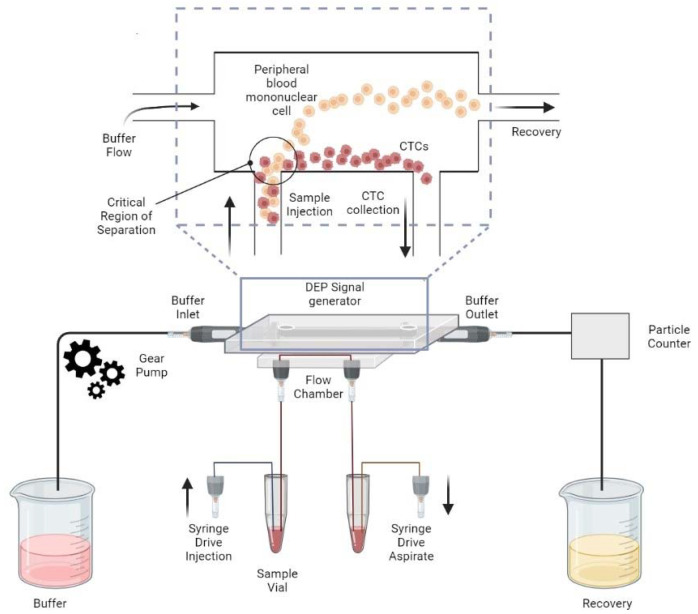
Side view schematic of the ApoStreamTM device showing the complex multi-inlet, multi-outlet and microfluidic system.

The most advanced DACS system, Apostream™ (Precision Medicine Group, LLC, Bethesda Metro Center #2, Bethesda, MD 20814, USA) (Figure 4), is based on a complex continuous field-flow DEP fractionation scheme (FFF-DEP), which uses planar interdigitated electrode configurations that cause exponentially decaying DEP forces [69]. ApoStream^TM^ is currently the most established DEP platform, enabling downstream enumeration and characterization of all CTCs from whole blood using laser capture cytometry in blood samples from patients with various solid tumors to capture epithelial and mesenchymal and stem CTCs (NSCLC adenocarcinoma, breast cancer, ovarian cancer and squamous cancer patients of the lung) [69,70].

The main limitation of ApoStream ™, as with similar DEP-based sorting systems, is the use of planar and interdigitated electrode configurations, which cause an exponential decay in the DEP forces. This limits the size of the sorting volume to which DEP forces can act efficiently and reduce cellular throughput, increasing sample processing to more than 10 h [29].

## 4. Conclusions

CTPCs are highly informative about both intra and extramedullary disease at the phenotypic, genomic and transcriptomic levels. Enumerating CTPCs is an emerging prognostic factor in MM, challenging their longitudinal monitoring in peripheral blood using a cheap and label-free emerging technology. Despite DEP being a young technique in the diagnostic field, continuous technological progress is ongoing to warrant fundamental discrimination implemented on electronic factors, such as membrane capacity and cytosol composition.

Due to the technical difficulties associated with the high-frequency actuation and the high sensitivity of the DEP response to small-cell variation within the cell subpopulation, the separation of cells based on *f_CO_*_2_, generally referred to as ultra-high-frequency DEP, has not been extensively explored as compared with low-frequency, *f_CO_*_1_-based DEP. Extending DEP measurements to include analyses of both frequencies *f_CO_*_1_ and *f_CO_*_2_ could enhance characterization of the dielectric properties of CTPCs, providing a novel tool to enrich cells of interest without labelling requirements and enable more efficient manipulations of cells (e.g., subpopulation enrichment or selective separation), not yet tested and validated in MM patients. In the next few years, the ever-major translational collaboration of different scientific researchers, such as engineers, physicists, clinical biochemists and molecular biologists, will improve label-free techniques, such as DEP, for CTPC isolation and longitudinal monitoring.

## Figures and Tables

**Table 1:** Current available and relative pros and cons, platforms to detect CTCs and cfDNA in peripheral blood of patients affected by multiple myeloma.

Platform	Pros	Cons
Multicolour Flow Cytometry (MCF)	- The ability to use multiple fluorescent markers at the same time allows for the identification of multiple cell types as well as functional markers that characterize each sample further. - Simultaneously analysis of over 20 parameters at a time.	- If fluidic instability occurs during sample acquisition, scatter and fluorescence sensitivity may be lost. - Because of the nonspecific sticking of antibodies, dead cells can masquerade as false positives.
Next-Generation Flow Cytometry (NGF)	- A highly valuable method for monitoring minimal residual disease (MRD) and determining the depth of complete response (CR) in multiple myeloma (MM) bone marrow (BM) after therapy. - Analytic Sensitivity of 0.0001%.	- Lower sensitivity than molecular methods. - Lacks standardization.
Next-generation sequencing (NGS)	- Comprehensive genomic coverage. - Ability to sequence hundreds to thousands of genes or gene regions simultaneously. - Easily interpretable results and potential for extremely high sensitivity.	- Sensitivity depends on the cellular input. - Not suggested to clinicians for decision-making.
GeneScanning	- Possibility of automation for high-throughput experiments. - High accuracy and analysis of DNA sequence up to 1000 bp.	- Under-representation or even complete absence of certain loci.

## Data Availability

Not applicable.
